# Observation of Extensive Chromosome Axis Remodeling during the “Diffuse-Phase” of Meiosis in Large Genome Cereals

**DOI:** 10.3389/fpls.2017.01235

**Published:** 2017-07-13

**Authors:** Isabelle Colas, Benoit Darrier, Mikel Arrieta, Sybille U. Mittmann, Luke Ramsay, Pierre Sourdille, Robbie Waugh

**Affiliations:** ^1^Cell and Molecular Sciences, The James Hutton Institute Dundee, United Kingdom; ^2^Institut National de la Recherche Agronomique UMR 1095, Génétique, Diversité & Ecophysiologie des Céréales Clermont-Ferrand, France; ^3^Université Clermont Auvergne–UBP Aubière, France; ^4^Division of Plant Sciences, University of Dundee at The James Hutton Institute Dundee, United Kingdom

**Keywords:** ASY1, synapsis, cereal, meiosis, chromatin

## Abstract

The production of balanced fertile haploid gametes requires the faithful separation of paired (synapsed) chromosomes toward the end of meiotic prophase I (desynapsis). This involves the timely dissolution of the synaptonemal complex during the pachytene-diplotene transition, a stage traditionally referred to as the “diffuse stage.” In species with large genomes such as, barley (*Hordeum vulgare* L.) and wheat (*Triticum aestivum* L.) we know most about the early stages of meiotic prophase I. There, synapsis initiates at the telomeric ends of chromosomes and progresses toward the centromeric regions through the ordered assembly of the synaptonemal complex (SC). Synapsis is impacted by recombination (crossing over, CO) which locally modifies the extent of chromatin compaction and extension. CO is uneven along the chromosomes, occurring mainly toward the telomeric regions resulting in a highly skewed distribution of recombination events. However, we know very little about the process of desynapsis which occurs during the “diffuse stage,” where the synapsed and recombined chromosomes faithfully desynapse and separate into daughter cells. Here, using 3D-SIM super-resolution immuno-cytology combined with the use of antibodies directed against two crucial SC proteins, ASY1 and ZYP1, we followed the whole of meiosis I (i.e., both synapsis and desynapsis) in both barley and wheat. We showed that synapsis forms a characteristic tri-partite SC structure in zygotene (more clearly seen in barley). Toward the end of meiosis I, as the SC starts to disassemble, we show that extensive chromosome axis remodeling results in the formation of characteristic “tinsel-like” structures in both wheat and barley. By using a mutant (*des10*) that is severely compromised in polymerization of ZYP1during synapsis, we show that tinsel structure formation during SC dissolution is not dependant on full synapsis and may relate instead to changes in expansion stress. Our observations highlight a potentially new role for ASYNAPSIS1 (ASY1) in desynapsis, in addition to chromosome synapsis and cohesion.

## Introduction

**Figure 1:**
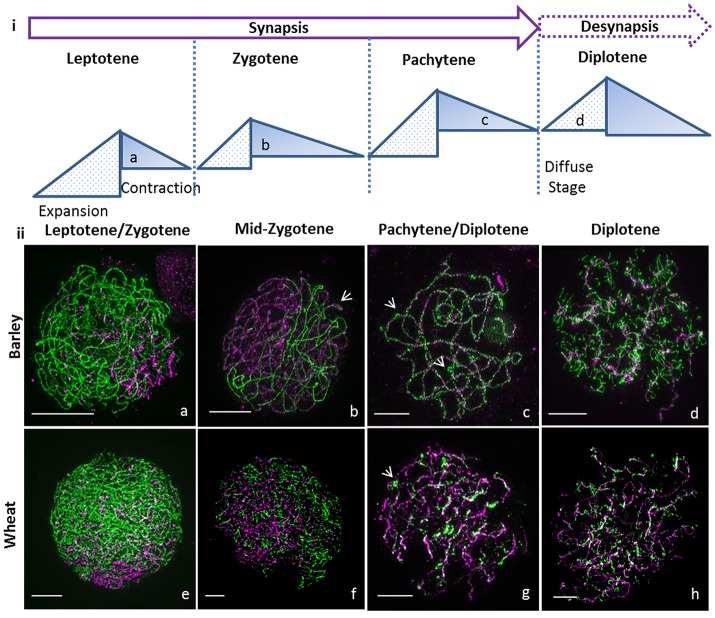
Super resolution microscopy of synapsis in large genome cereals. **(i)** Cartoon of cycle of chromatin expansion and contraction as described in Kleckner et al. (2004) with indication of the diffuse stage. **(ii)** Synapsis was monitored using ASY1 (green) and ZYP1 (magenta) by 3D-SIM for barley Bowman **(a–d)** and wheat Chinese Spring **(e–h)**. Synapsis starts in leptotene at one end of the nucleus **(a,e)** and ZYP1 polymerizes to bring the chromosomes together during zygotene **(b,f)** though the tripartite structure of the synaptonemal complex is only visible in barley wt (**b**, arrow). At pachytene, synapsis is complete in barley **(c)** and wheat **(g)** with ectopic ASY1 signals (**c**,**g**, arrows). During diplotene, ASY1 remodels in both species **(d,h)** to form tinsel structures. Scale bar 5 μm.

Meiosis is a pair of specialized cell divisions (meiosis I and II) that are required for the formation of parental gametes prior to fertilization (Zickler and Kleckner, 1999; Zamariola et al., 2014). The process involves profound changes in chromosome structure and organization and is both tightly regulated and mechanistically conserved between plants and animals (Kleckner et al., 2004; Gerton and Hawley, 2005). During meiosis I, homologous chromosomes pair and then synapse through formation of the proteinaceous synaptonemal complex (SC; Zickler, 2006) that is coordinated with inter-chromosomal recombination (crossing over, CO) where genetic material is exchanged (Mercier et al., 2014). Prior to the formation of the SC, proteins such as, ASYNAPTIC 1 (ASY1) organize the chromosome axes by interacting with chromatin to form lateral elements as early as leptotene. Lateral elements of each homolog are then brought together during zygotene by the formation of the central element comprising proteins that include ZIPPER-LIKE 1(ZIP1). Toward the end of meiosis I, homologous chromosomes that are paired all along their length subsequently need to separate and divide faithfully into daughter cells. This occurs by dissolution of the SC, during the transition from pachytene to diplotene (that includes the cytologically defined “diffuse stage”) with the sites of CO physically holding homolog together and orienting chromosomes prior to division (Zickler and Kleckner, 1998, 1999; Zickler, 2006; Mercier et al., 2014). It has also been recognized that during synapsis recombination events impact on the compaction and extension of local chromatin, imposing physical constrains on the axes (Figure 1i; Kleckner et al., 2004; Higgins et al., 2012). While SC assembly has been studied extensively, due to a combination of technical and biological barriers SC dissolution during the diffuse stage has not. Aberrations in SC dissolution are however deleterious and can lead to chromosome mis-segregation and loss of the genetic integrity (Sanchez-Moran et al., 2007; Joyce and McKim, 2010; Kim et al., 2014).

Here, in both barley and wheat, using super-resolution immuno-cytology (3D-SIM) with antibodies against ASY1 (Armstrong et al., 2002) and the SC protein ZYP1 (Barakate et al., 2014) we reveal details of chromosome organization during meiosis I, that cannot be seen using confocal imaging (Supplementary Figures 1, 2). We observe that the axis protein ASY1, canonically considered only to be involved in the early stage of meiosis, persists through desynapsis where it reveals characteristic and transient “tinsel-like” physical structures. These dynamic changes in chromosome structure are correlated with sequential rounds of chromatin expansion and contraction and could thus possibly be due to mechanical stress and stress relief, respectively (Kleckner et al., 2004; Higgins et al., 2012) as proposed in a mechanical model of chromosome function (Kleckner et al., 2004).

## Results

### ASY1 reveals a novel structure at diplotene in large cereal genomes

At leptotene, synapsis initiates at one side of the nucleus in the telomeric regions (Colas et al., 2008; Higgins et al., 2012) in both barley (Figure 1iia) and wheat (Figure 1iie, Supplementary Figure 3, and Supplementary Video 1), progresses along the chromosomes, and most obviously in barley, forms a characteristic tri-partite SC structure in zygotene (Figure 1iib, arrow, Supplementary Figure 3, and Supplementary Video 2). The visualization of the tri-partite structure in wheat was more problematic (Figure 1iif, Supplementary Figure 3, and Supplementary Video 3). At pachytene, in barley and wheat the chromosomes are fully synapsed and coiled (Figures 1iic,g, Supplementary Figure 3, and Supplementary Videos 4, 5) and we noted bright ASY1 signals on the surface of the SC (Figures 1iic,g arrow) that potentially represents the first step in chromosome condensation and desynapsis.

At diplotene, in both species we observed that the ASY1 axis (Figures 1iid,h, Supplementary Figure 3, and Supplementary Videos 6, 7) dynamically re-organizes into transient structures that superficially resemble lampbrush chromosomes (LBCs) observed in many animal oocytes during the prolonged resting diplotene (dicyate; Morgan, 2002). We named these novel and previously undescribed physical forms “tinsel-likestructures” given their resemblance to the popular Christmas decoration.

### ASY1 re-organization is not dependent on normal synapsis

**Figure 2:**
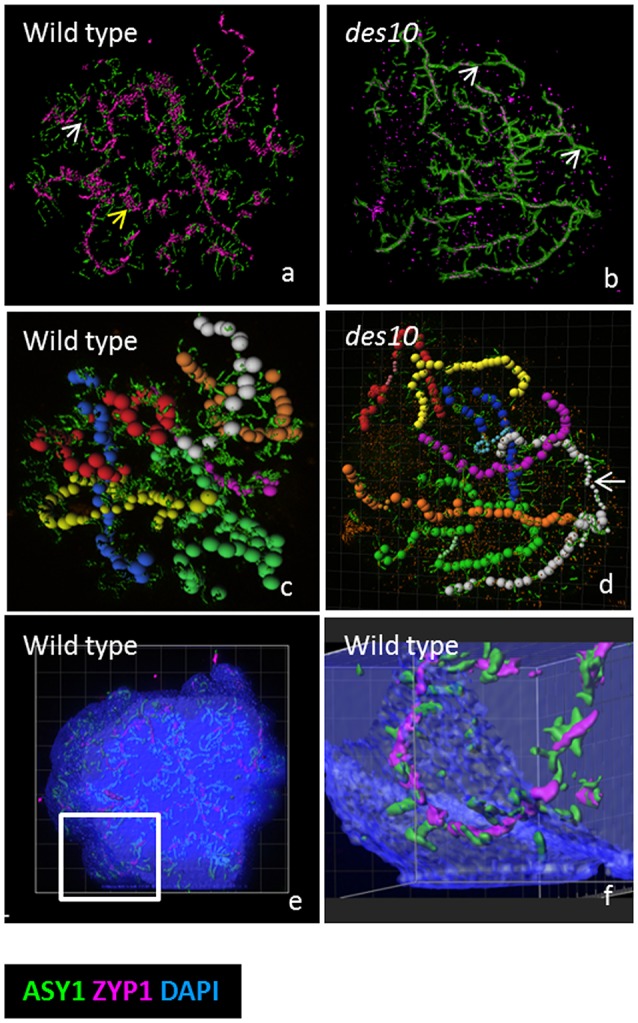
Modeling of the tinsel structure in wt and *des10*. Modeling the tinsel structure in wt **(a)** and *des10* **(b)** by creating “surfaces” for ASY1 and ZYP1 using Imaris surface tool **(a,b)**. This revealed areas of axes with no ZYP1 (white arrow) in both wild type **(a)** and *des10* **(b)**, suggesting dissolution of the SC. ZYP1 aggregates were visible in wild type (a, yellow arrow), with abundant surrounding ASY1, that could suggest contraction of local chromatin. Individual bivalents are manually tracked using the Imaris measurement tool **(c,d)** revealing different thickness of bivalent areas in *des10* (d, white arrow), that may suggest differences in the thickness of the remnant ASY1 axes. Using the tool surface on DAPI channel (with 50% transparent effect to view inside the DAPI signal) revealed that ASY1 remodeling remains within the chromatin **(e,f)**.

To test whether a perturbed synapsis would impact on this striking distribution of ASY1 at diplotene, we then examined the barley *desynapsis10* meiotic mutant (*Des10* = *HvMlh3*) in which normal synapsis and chromatin organization are (Colas et al., 2016). Early prophase I in *des10* appears cytologically normal while at later stages, polymerization of ZYP1 is severely compromised (Colas et al., 2016). Despite this obvious impediment, *des10* mutants form tinsel-structures during SC dissolution (Figures 2a,b, and Supplementary Video 8), though their organization is distinct from wild type. It is possible to track individual bivalents at the tinsel-structure stage in both wild type and *des10* (Figures 2c,d) and it is evident that the ASY1 signals re-organize within the chromatin (Figures 2e,f and Supplementary Figure 4). Thus, the tinsel-like structures are bound within the chromatin of the chromosomes and while they are only fully elaborated when synapsis is complete, their formation in *des10* implies that the dissolution of SC is not dependent on full synapsis, and may relate instead to changes in expansion stress.

### Organization of ASY1 along the axes changes during desynapsis

**Figure 3:**
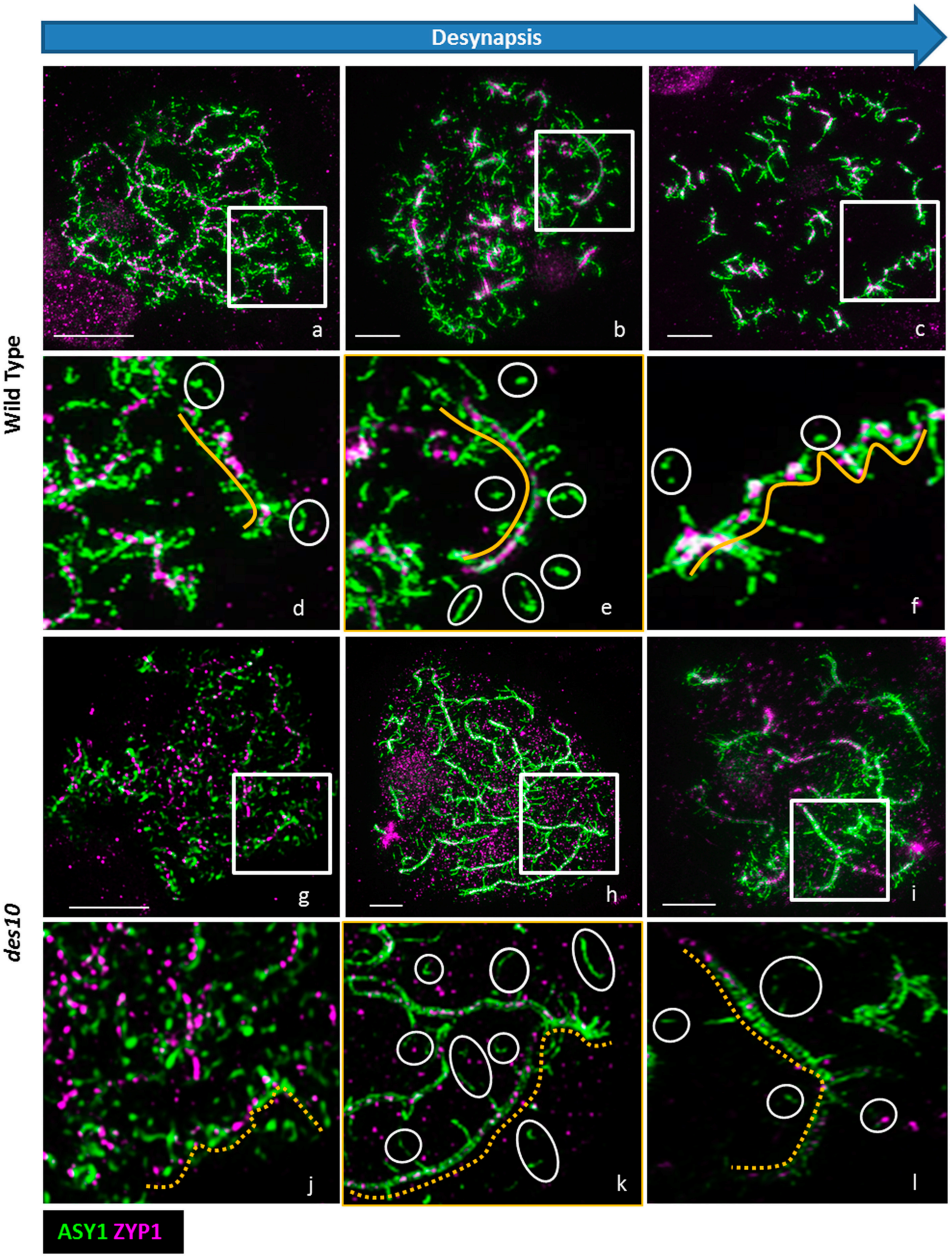
ASY1 re-organization during desynapsis. De-synapsis was monitored with 3D-SIM using ASY1 (green) and ZYP1 (magenta) in barley wild type **(a–f)** and *des10* **(g–l)**. Enlarged areas of wild type **(d–f)** and *des10* **(j–l)** slides show the organization of ASY1 along the ZYP1 axes (yellow lines). White circles highlight isolated ASY1 signals **(d–f)** that may represent the process of dissolution of ASY1 after the tinsel structure organization. In *des10* **(j–l)**, ZYP1 axes are not linear (dashed yellow lines), but ASY1 displays a similar behavior to wild type.

In later stages of diplotene, during the contraction phase (Figure 1i; Kleckner et al., 2004), the loops and stretches containing ASY1 that are visible at early diplotene (Figures 3a–c) become separated from the core lateral element of the SC (Figures 3d–f, yellow lines), that itself becomes depleted of ASY1 (Figures 3d–f white circles). In *des10*, ASY1 also re-organized as loops and stretches, but some differences from wild type were observed (Figures 3g–l) so that even at early diplotene, it was not possible to see the tri-partite SC structure as in the wild type (Figures 3g,h). Instead, the *des10* tinsel-structures appeared cytologically similar to those in wheat (Figure 1iih; though with less ZYP1 signal). Progression is similar to wild type though with, the core lateral element of the SC in *des10* (Figures 3j–l), yellow dash lines) becoming depleted of ASY1 (Figures 3k,l, white circles).

### ASY1 in meiosis II and chromosome segregation

**Figure 4:**
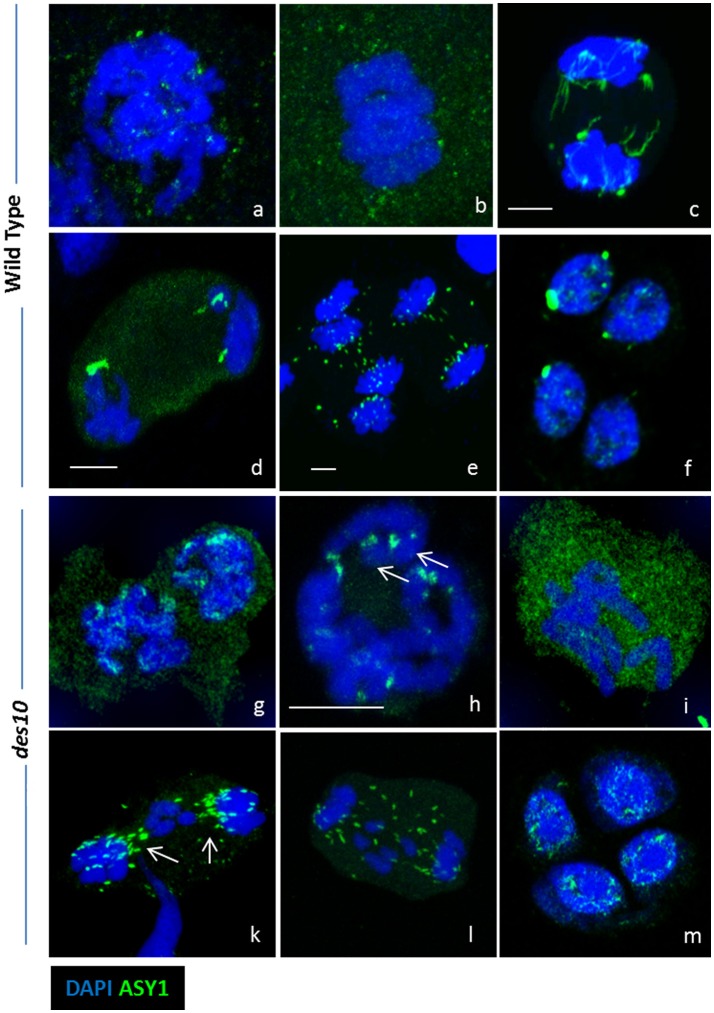
ASY1 re-organization during desynapsis and chromosome segregation. Strong ASY1 signal are detected after prophase I during chromosome segregation in both wild type **(a–f)** and *des10* **(g–m)**. Discrete ASY1 foci (green) are visible in wild type diplotene **(a)**. At metaphase I, ASY1 signal does not seems specific and is located in the cytoplasm **(b)**. During anaphase I, ASY1 signal is either linear **(c)** or globular **(d,e)**. Discrete ASY1 signals are also found in tetrads **(f)**. ASY1 behaves the same in *des10*, but the delay in prophase enables more details to be studied. ASY1 signal is present on diplotene chromosomes **(g)**, and it is possible to visualize the last ASY1 signal near the end of the chromosomes (**h**, arrow). During metaphase I, ASY1 is mainly in the cytoplasm **(I)** but during anaphase I, it is possible to see the ASY1 signal relocated around the lagging chromosomes (**k,l** arrows). Discrete ASY1 signal is also found in tetrads **(m)**. Scale bar 5 μm.

A potential role for ASY1 within the tinsel structures is supported by the protein's persistence into later stages of meiosis I (Figure 4). In barley WT, we detect discrete ASY1 foci on late diplotene chromosomes and in the cytoplasm (Figure 4a). At metaphase I, as expected, no discrete ASY1 signal was detectable on the chromosomes but was detected in cytoplasm of WT (Figure 4b) suggesting that ASY1 is either being degraded or has diffused throughout the nucleoplasm. However, strong ASY1 signals reappeared in WT at anaphase I during chromosome segregation (Figures 4c–e) adopting linear (Figure 4c) or globular conformations (Figures 4d,e). The globular ASY1 signal is also detected at the tetrad stage (Figure 4f). Similarly, in *des10*, discrete ASY1 foci could be detected on late diplotene chromosomes and in the cytoplasm (Figure 4g). Moreover, due to the *des10* having a longer prophase (Colas et al., 2016), it was possible to see the separation of the bivalent ends (telomeric regions) resulting from a defect in chiasmata formation (Figure 4h) and detect ASY1 signal in the same region (Figure 4h, arrow). At metaphase I (Figure 4i), *des10* behaved as in the wild type with no detectable ASY1 signal on the chromosomes, but a considerable amount in the cytoplasm. However, strong ASY1 signals reappeared in *des10* anaphase I during chromosome segregation (Figures 4h,l) that later become concentrated at the telomeric regions (Figure 4k, arrow) as if following microtubule orientation (Cabral et al., 2014).

## Discussion

In this study, we compared the progression of synapis, and desynapsis, in barley and wheat and observed notable differences between the two organisms. Although, behaving broadly similarly, in wheat it was not always possible to observe the tri-partite structure of the SC. We note that it has been previously reported that wheat prophase is shorter than in barley despite the wheat genome being three times the size of the barley genome (Bennett and Finch, 1971; Bennett et al., 1971; Bennett, 1984). This suggests that in wheat, the chromatin compaction/extension during prophase could be faster than in barley, leading to chromatin appearing more compacted during analysis, which could explain why the SC tripartite structure was not easily recognizable in wheat.

We have previously shown that in the barley meiotic mutant *des10*, the chromatin compaction/extension cycle was perturbed (Colas et al., 2016). As a consequence, synapsis in wheat and *des10* superficially appear to be similar as it was also not always possible to observe the tri-partite structure in *des10*. Therefore, it is possible that the absence of obvious tri-partite structure in both wheat and *des10* are due to higher chromosome coiling and chromatin compaction (Colas et al., 2016).

ASY1 belongs to the HORMA domain protein family which includes HOP1 (yeast), PAIR2 (rice), HIM3 (*C. elegans*) and HORMAD1/2 (mouse; Zetka et al., 1999; Nonomura et al., 2006; Boden et al., 2009; Wojtasz et al., 2009). In mouse, HORMAD1, becomes depleted from the synapsed chromosome axis, only to reappear at diplotene (Wojtasz et al., 2009) and HORMA domain proteins have also been shown to associate with chromosomes during the later stages of meiosis in both mouse (Shin et al., 2010) and *C. elegan* (Zetka et al., 1999). Moreover, in the rice PAIR2 HORMA domain protein mutant (orthologous to ASY1) segregating chromosomes at anaphase I are improperly orientated on the spindle and are often transversal to the microtubules (Nonomura et al., 2006). Similarly in wheat, *TaASY1* RNAi (reduced ASY1 expression), chromosomes are improperly orientated at metaphase I (Boden et al., 2009). It is worthwhile noting that these *TaASY1* RNAi lines exhibit residual ASY1 gene expression, and that some ASY1 could therefore still load onto the chromosomes and polymerize. However, the ASY1 signal was very faint at diplotene, and, it is virtually impossible to recognize any sort of tinsel-like coiled structure. In the same study, confocal images of the *Ph1b* deletion line exhibit the very same coiling patterns (Supplementary Figure 4), that we can now attribute to the tinsel-like structure. A recent study in rye also revealed some ASY1 coiling under confocal images, resembling our own observations (Simanovsky et al., 2014). We conclude that tinsel-structures represent transient structures that appear during SC dissolution and that may be specific to large cereal genomes.

Superficially there appears to be some similarities with the well-described Lampbrush chromosomes observed in oocytes during an extended diplotene. Lampbrush chromosomes are associated with intense transcriptional activity, presumably as a precursor to the massive synthesis of new proteins required for subsequent oocyte development (Morgan, 2002). Their appearance is correlated with the “diffuse stage of chromatin” (Klasterska, 1976, 1978), which has been shown to have high transcriptional activities in in large genome species with extended diplotene stage (Kolowerzo-Lubnau et al., 2015). By definition this is hard to see in barley, but the diffuse stage is considered to be the phase when the chromatin has become relaxed in order to enable the transition from pachytene to diplotene (Stack and Anderson, 2001; Zhang et al., 2008), a key stage in preparing chromosomes for desynapsis and segregation. While several previous observations have suggested that plants could also display lampbrush chromosomes, the supporting data remain somewhat inconclusive (Grun, 1958; Spring et al., 1975). Lampbrush chromosomes are observed in a range of taxa are very specific morphological features and they appear to be somewhat different from the unique and temporally re-organized SC structures we observe during desynapsis. We are mindful that due to the presence of the cell wall in plant tissues, cytological techniques in general tend to be quite harsh and this could potentially affect our ability to detect classical “lampbrush-like” structures (Klasterska, 1978). However, the protocol we adopt is gentle, helping preserve the 3D structure of the nucleus and thus we have called the structures we observe “tinsel-structures” to avoid confusion.

The persistence of ASY1 in later stages of meiosis I in barley is surprising and though it is possible that this could represent residual protein aggregated in the nucleus, it seems improbable given the loss at metaphase I and subsequent reappearance. The presence of ASY1 found here also mirrors its recently reported cytological persistence in wheat (Sepsi et al., 2016), indicating that our observations of ASY1 in tinsel structure and desynapsis may form part of an extended functional role late in meiosis I.

Thus, in wheat and barley ASY1 may have a role in chromosome orientation, as indicated in rice (Nonomura et al., 2006) and reported for the *C. elegans* ortholog HIM-3 (Zetka et al., 1999). We note it is also consistent with ASY1 expression detected in the later stages of meiosis in both Arabidopsis and wheat, that was discounted as being due to the presence of tissues at earlier meiotic developmental stages within the samples used to extract RNA (Armstrong et al., 2002; Boden et al., 2009).

## Conclusions

We conclude that desynapsis is highly coordinated in the large genome cereals barley and wheat, and involves the formation of novel “tinsel-like” structures in which ASY1 appears to play a major role. The assembly and dissolution of these structures can be explained in the terms of a canonical model of chromosome function based on mechanical forces (Kleckner et al., 2004), and potentially relates to previous cytological observations at diplotene in a range of large genome plants (Zhang et al., 2008; Boden et al., 2009; Simanovsky et al., 2014) that may relate to the lampbrush chromosomes found in animal oocytes (Morgan, 2002). Our use of a protocol devoid of strong denaturing treatments combined with the resolution of 3D-SIM facilitated more detailed visualization of the diffuse stage, which in turn led us to establish the hypothesis that ASY1 plays an important role in desynapsis. This role relates to observations of its persistence into later stages of meiosis I and would be consistent with a broader function in chromosome segregation, potentially through the mediation of mechanical expansion stress. Given the lack of an obvious pachytene check-point in plants, unlike other systems (Li et al., 2009), our elucidation of chromosome structures during the pachytene-diplotene transition provides an unique platform to study the control of desynapsis.

## Materials and methods

### Plant and material preparation

The plants used in this study were the barley cv. Bowman, Bowman near isogenic line BW230 (*des10*), and hexaploid wheat cv. Chinese Spring. Plants were grown at 20°C for 16 h light and 15°C for 8 h dark until they reached meiosis. Anthers were staged according to Colas et al. (2008, 2016).

### Immunocytology

Plant material was fixed and prepared according to Colas et al. (2016). We used TaASY1 rabbit antibody and custom HvZYP1 rat antibody (Dundee Cell Product) at 1:2,000 and 1:500, respectively. We used secondary antibodies consisting of a mixture of anti-rabbit Alexa Fluor® (488 or 568) and/or anti-rat Alexa Fluor® (568 or 488; Life Technologies) diluted in 5% donkey/goat serum in 1xPBS, 0.5% Triton™ X100 blocking solution (1:600). Slides were washed in 1xPBS, counterstained with DAPI and mounted in Vectashield® (H-1000, Vectorlabs). Vectashield containing DAPI could also be used.

### Microscopy

3D Confocal stack images (512 × 512, 12 bits) were acquired with LSM-Zeiss 710 using laser light 405, 488, and 561 nm sequentially with 4 lines averages. Projections of 3D pictures and light brightness/contrast adjustment were performed with Imaris 8.0.2 (Bitplane). 3D-SIM images were acquired on a DeltaVision OMX Blaze (GE Healthcare) for Laser light 405, 488, and 564 nm as described in Colas et al. (2016). Super-resolution three-dimensional image stacks were reconstructed with SoftWorx 6.0 (GE). 3D projection and surface modeling were performed with Imaris 8.0.2 (Bitplane)

## Author contributions

IC, LR, and RW designed the study. IC, BD, MA, and SM carried out experiments and analysis. IC, LR, PS, and RW wrote the paper.

### Conflict of interest statement

The authors declare that the research was conducted in the absence of any commercial or financial relationships that could be construed as a potential conflict of interest.

## Supplementary material

Supplementary Figure 1 Confocal images of synapsis in barley. Synapsis of homologous chromosomes (ASY1 labelled, green) is followed by the polymerization of ZYP1 (magenta) for leptotene **(a–e)**, zygotene **(f–j)**, pachytene **(k–o)** and diplotene **(p–t)**. ASY1 labelling at diplotene **(q,s)** (dissolution of synapsis) is very fragmented and patchy. Scale bar 5 μm.

Supplementary Figure 2 Confocal images of synapsis in wheat. Synapsis of homologous chromosomes (ASY1 labelled, green) is followed by the polymerization of ZYP1 (magenta) for G2 **(a,f,k,p)**, leptotene **(b,g,l,q)**, mid-zygotene **(c,h,m,r)**, late zygotene **(d,I,n,s)** and diplotene **(e,j,o,t)**. ASY1 labelling at diplotene **(o,t)** (dissolution of synapsis) is very fragmented and patchy. Scale bar 5 μm.

Supplementary Figure 3 OMX details of Figure 1. Synapsis is followed in detail with 3D-SIM using ASY1 (green) and ZYP1 (magenta) for barley **(a–h)** and wheat **(i–p)**. Synapsis starts at leptotene **(a,e)**. In barley the tripartite structure of the SC becomes visible at zygotene (**b,f**, arrow). At late pachytene, small loops of ASY1 are evident (**c,g**, arrow), that may represent the initial formation of the larger loops at later stages (**d,h**, arrow). In wheat, synapsis start similarly to barley **(I,m)** but the SC tri-partite structure is not obvious along the bivalents **(j,n)** but is partially evident during pachytene (**k,o**, arrow). At diplotene **(l,p)**, wheat also forms tinsel like structures. White boxes indicate zoomed area.

Supplementary Figure 4 Gallery image of wild type barley tinsel structure. The DAPI (blue) labeled chromatin shows the individual thick bivalents during diplotene. When navigating through the section, we can see that ASY1 (green), ZYP1 (magenta) are embedded within the DAPI signal and thus within the chromatin.

Supplementary Video 1 3D wheat Leptotene with ZYP1 (magenta) and ASY1 (green).

Supplementary Video 2 3D Barley Zygotene with ZYP1 (magenta) and ASY1 (green).

Supplementary Video 3 3D Wheat Zygotene with ZYP1 (magenta) and ASY1 (green).

Supplementary Video 4 3D Barley Pachytene with ZYP1 (magenta) and ASY1 (green).

Supplementary Video 5 3D wheat Pachytene with ZYP1 (magenta) and ASY1 (green).

Supplementary Video 6 3D Barley Tinsels with ZYP1 (magenta) and ASY1 (green).

Supplementary Video 7 3D wheat Tinsels with ZYP1 (magenta) and ASY1 (green).

Supplementary Video 8 3D *des10* Tinsels with ZYP1 (magenta) and ASY1 (green).
