# Supplementary figures and images for: Observation of Extensive Chromosome Axis Remodeling during the “Diffuse-Phase” of Meiosis in Large Genome Cereals
Source: Front Plant Sci. 2017 Jul 13;8:1235. doi: 10.3389/fpls.2017.01235 (PMC5508023; doi:10.3389/fpls.2017.01235)

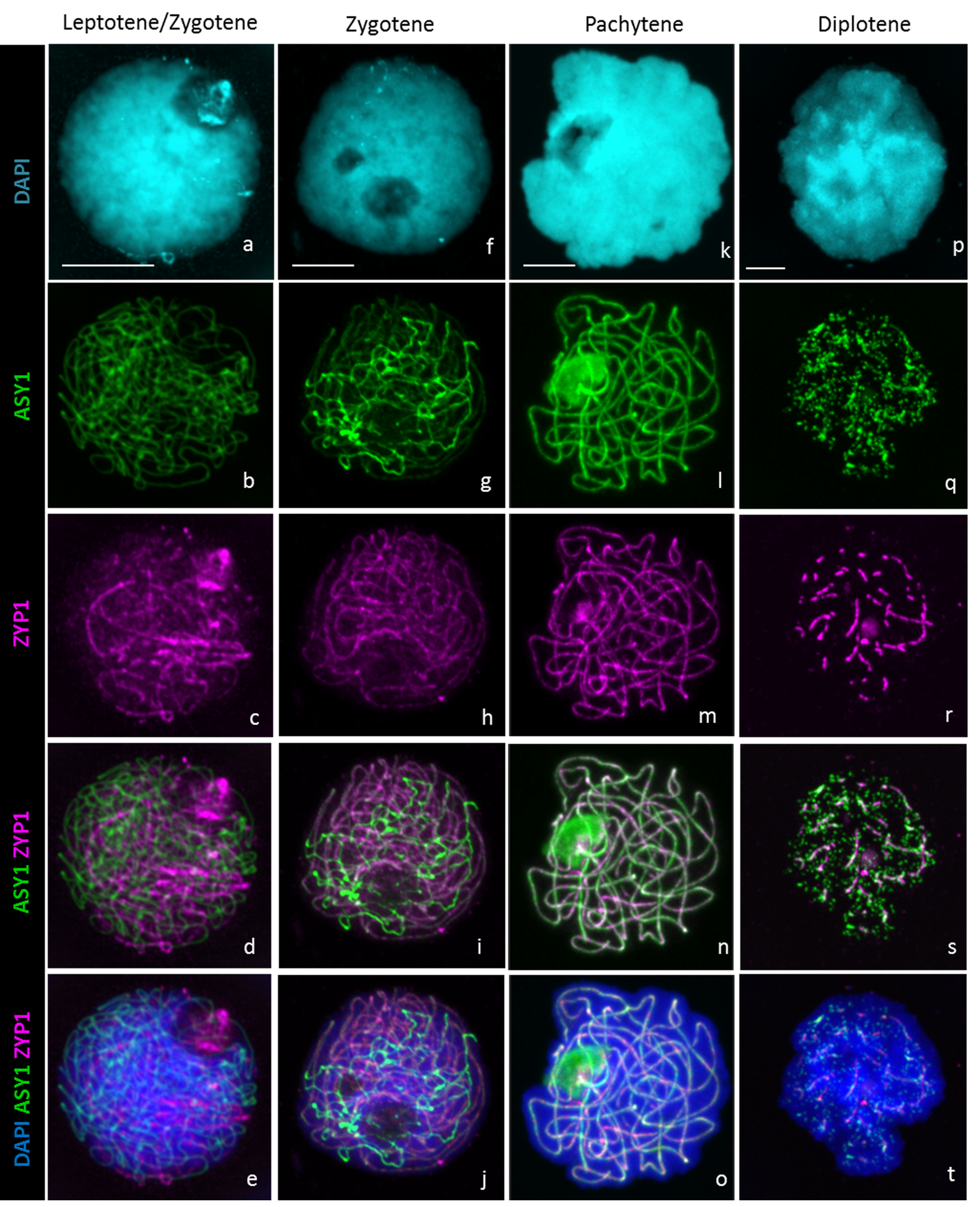

Supplement: Supplementary Figure 1 — Confocal images of synapsis in barley. Synapsis of homologous chromosomes (ASY1 labelled, green) is followed by the polymerization of ZYP1 (magenta) for leptotene (a–e), zygotene (f–j), pachytene (k–o) and diplotene (p–t). ASY1 labelling at diplotene (q,s) (dissolution of synapsis) is very fragmented and patchy. Scale bar 5 μm. [file Image1.TIF]

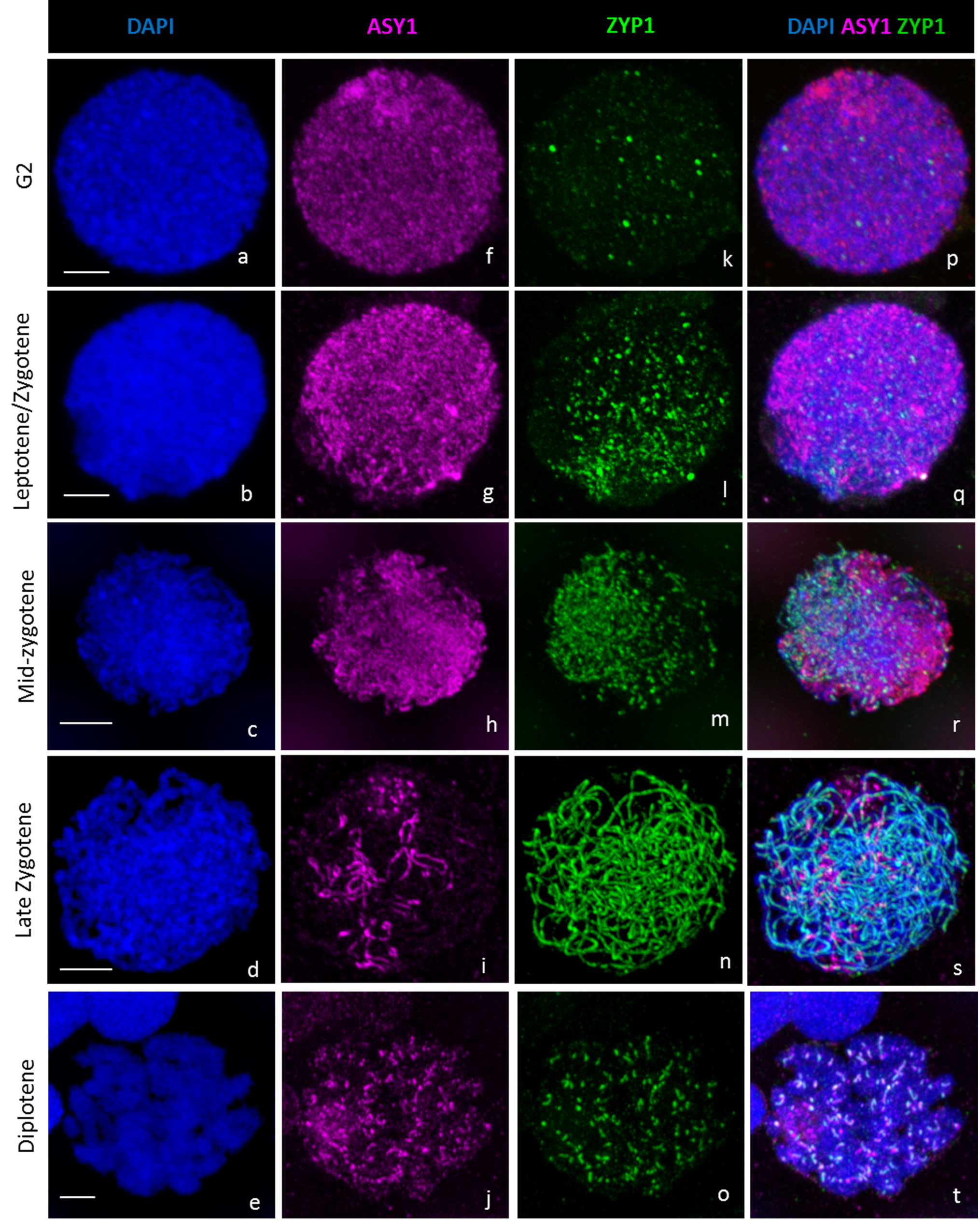

Supplement: Supplementary Figure 2 — Confocal images of synapsis in wheat. Synapsis of homologous chromosomes (ASY1 labelled, green) is followed by the polymerization of ZYP1 (magenta) for G2 (a,f,k,p), leptotene (b,g,l,q), mid-zygotene (c,h,m,r), late zygotene (d,I,n,s) and diplotene (e,j,o,t). ASY1 labelling at diplotene (o,t) (dissolution of synapsis) is very fragmented and patchy. Scale bar 5 μm. [file Image2.TIF]

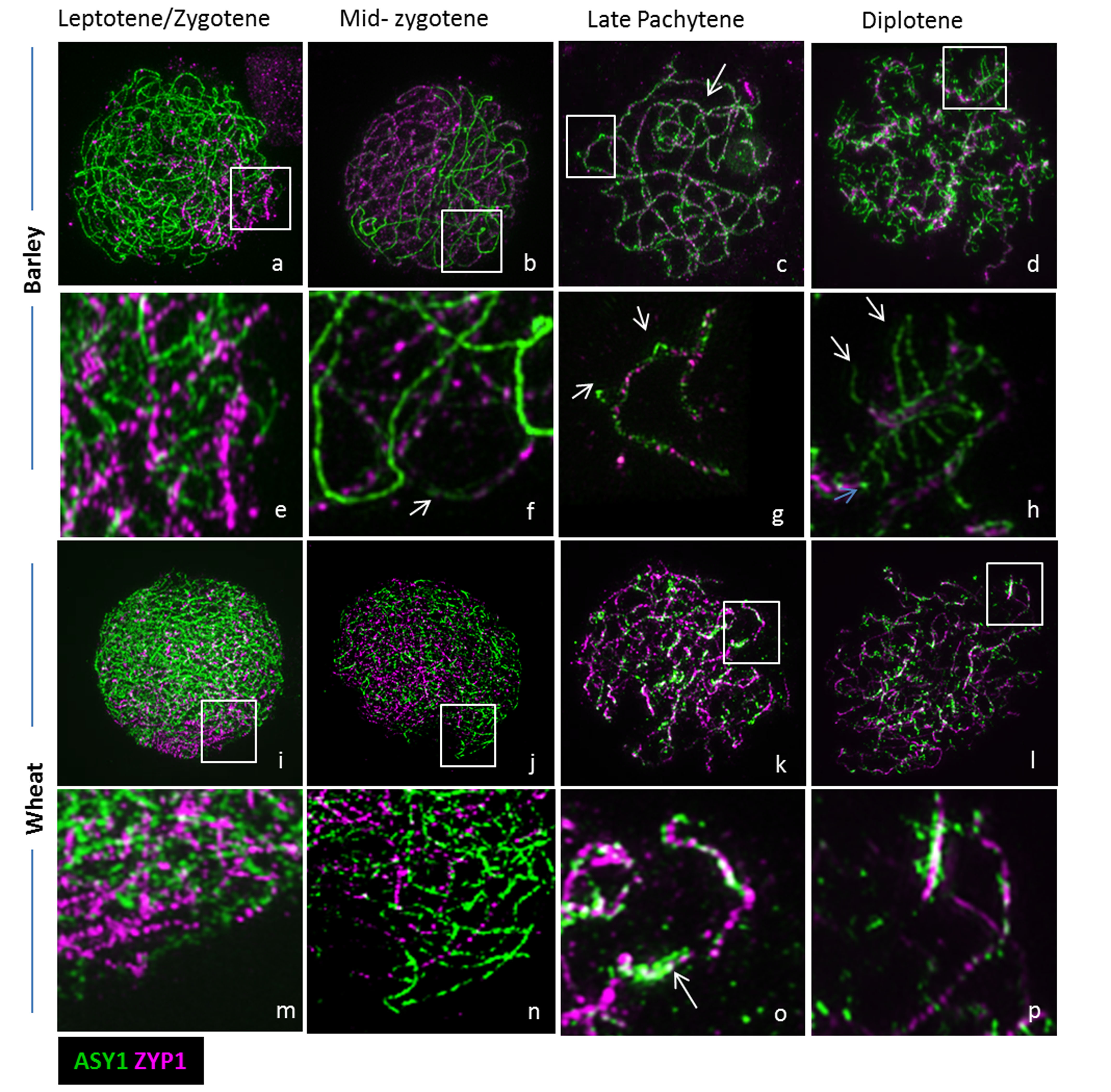

Supplement: Supplementary Figure 3 — OMX details of Figure 1. Synapsis is followed in detail with 3D-SIM using ASY1 (green) and ZYP1 (magenta) for barley (a–h) and wheat (i–p). Synapsis starts at leptotene (a,e). In barley the tripartite structure of the SC becomes visible at zygotene (b,f, arrow). At late pachytene, small loops of ASY1 are evident (c,g, arrow), that may represent the initial formation of the larger loops at later stages (d,h, arrow). In wheat, synapsis start similarly to barley (I,m) but the SC tri-partite structure is not obvious along the bivalents (j,n) but is partially evident during pachytene (k,o, arrow). At diplotene (l,p), wheat also forms tinsel like structures. White boxes indicate zoomed area. [file Image3.jpg]

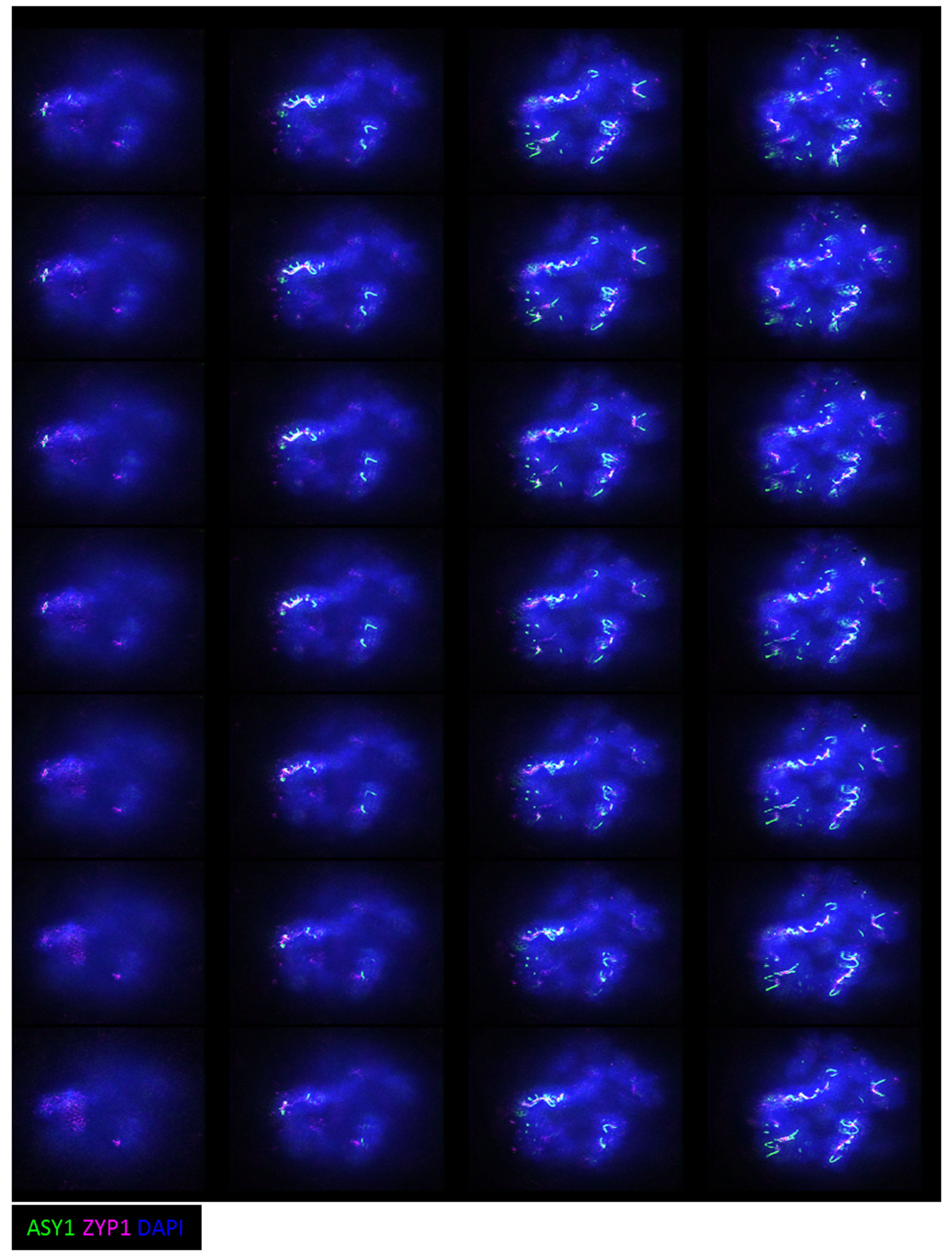

Supplement: Supplementary Figure 4 — Gallery image of wild type barley tinsel structure. The DAPI (blue) labeled chromatin shows the individual thick bivalents during diplotene. When navigating through the section, we can see that ASY1 (green), ZYP1 (magenta) are embedded within the DAPI signal and thus within the chromatin. [file Image4.jpg]
